# Microstructure Investigation of 13Cr-2Mo ODS Steel Components Obtained by High Voltage Electric Discharge Compaction Technique

**DOI:** 10.3390/ma8115381

**Published:** 2015-11-02

**Authors:** Igor Bogachev, Artem Yudin, Evgeniy Grigoryev, Ivan Chernov, Maxim Staltsov, Oleg Khasanov, Eugene Olevsky

**Affiliations:** 1 Material Science Department, Moscow Engineering Physics University, Moscow 115409, Russia; halfdeath0@gmail.com (A.Y.); eggrigoryev@mephi.ru (E.G.); i_chernov@mail.ru (I.C.); msstaltsov@mephi.ru (M.S.); eolevsky@mail.sdsu.edu (E.O.); 2 Department of Nanomaterials and Nanotechnologies, National Research Tomsk Polytechnic University, Tomsk 634050, Russia; khasanov@tpu.ru; 3 Department of Mechanical Engineering, San Diego State University, San Diego, CA 92182, USA

**Keywords:** field-assisted, powder consolidation, steel, oxide dispersion strengthening

## Abstract

Refractory oxide dispersion strengthened 13Cr-2Mo steel powder was successfully consolidated to near theoretical density using high voltage electric discharge compaction. Cylindrical samples with relative density from 90% to 97% and dimensions of 10 mm in diameter and 10–15 mm in height were obtained. Consolidation conditions such as pressure and voltage were varied in some ranges to determine the optimal compaction regime. Three different concentrations of yttria were used to identify its effect on the properties of the samples. It is shown that the utilized ultra-rapid consolidation process in combination with high transmitted energy allows obtaining high density compacts, retaining the initial structure with minimal grain growth. The experimental results indicate some heterogeneity of the structure which may occur in the external layers of the tested samples due to various thermal and electromagnetic in-processing effects. The choice of the optimal parameters of the consolidation enables obtaining samples of acceptable quality.

## 1. Introduction

Refractory materials, which are used in the fast reactor active zone, should meet a number of special requirements. However, many of the materials currently utilized in fuel claddings and other components of the reactor active zones do not meet the levels of the required mechanical properties, radiation, and thermal resistance, especially for the new projects on high-power nuclear reactors. They should have high thermal resistance at elevated temperatures, low thermal creep, low swelling, and low properties of degradation under irradiation. Also, they should possess suitable mechanical properties to withstand the pressure of a heat transfer agent and fuel. Corrosion resistance is also necessary. Currently, in many cases, special austenitic steels such as 16Cr-15Ni-2Mo steel are used as the material for cladding tubes for fast nuclear reactor active zones. These steels (with FCC crystal lattice) have suitable mechanical properties and radiation resistance at elevated temperatures and high neutron fluence [1,2]. However, in the next generation of fast reactors, for more energy efficiency the operating temperature will be higher, and the resulting neutron fluence will be higher due to the increase of the fuel campaign [2,3,4]. Alternatively, ferritic/martensitic steels with BCC lattice could be used for these applications because of their higher radiation resistance and, in particular, because of low swelling in comparison to austenitic steels. Yet these steels have quite high thermal creep at elevated temperatures which limits their use in the active zone [2,3,4]. To improve this parameter, oxide dispersion strengthening (ODS) of the matrix material by the refractory hard micro- or nanoscale particles is used. This approach enables obtaining thermal stability of the matrix steel, inhibiting dislocation movement and, as a result, decreases thermal creep [5,6,7,8,9,10,11,12]. Thereby, ODS steels are a prospective material for nuclear applications. The development of efficient production routes and obtaining suitable properties of the produced ODS materials are important modern problems whose solutions should enable the use of these steels in nuclear reactors. In most cases, ODS steels are produced via a powder processing route [5,6,7]; therefore, the mechanical properties of the fabricated ODS steel components become a major controlling factor.

The present work explores the possibility of a new method for the consolidation of ODS steel components by employing the high voltage electric discharge compaction technique. It allows obtaining homogeneous distribution of material components, controlling mechanical and physical properties of the final product at the stage of manufacturing, and achieving different final shapes of the product without any intermediate treatment [13,14,15]. It should be noted that electromagnetic field-assisted powder consolidation, such as spark plasma sintering, microwave sintering, *etc.*, has great advantages for the processing of such materials and, in particular, of oxide dispersion-strengthened steels. The high voltage electric discharge compaction (HVEDC) technology occupies a special place among the above-mentioned techniques. It has unique operation parameters in comparison with other electromagnetic field-assisted techniques [16,17,18].

High voltage electric discharge compaction technology has a number of advantages in comparison with conventional powder processing techniques such as hot extrusion, hot and cold isostatic pressing, and others. This method involves the uniaxial pressing of the powder in a non-conducting matrix with discharging a powerful pulse of electric current through the specimen. Electric energy of a set value is stored in a block of high-voltage capacitors and pressure is controlled by a pneumatic press.

Such an ultra-rapid process in combination with high transmitted energy allows obtaining high density compacts, saving the initial structure with minimal grain growth, and avoiding thermally activated phase transformations. All these parameters are important for the ODS steels compaction because there is a need to obtain homogeneous distributions of the hard refractory oxide particles inside the matrix steel powder to prevent their agglomeration and grain growth for better mechanical properties. Also, this manufacturing technology has the ability of final product net-shaping. Using various shapes of punches and dies, the freeform products can be manufactured.

## 2. Materials and Methods

The initial base material for the conducted investigations was ferritic/martensitic 13Cr-2Mo special reactor steel, with the chemical composition shown in Table 1.

**Table 1:** Chemical composition of the investigated steel powder.

C	Si	Mn	Cr	Ni	Mo	Nb	V
0.10–0.15	<0.6	<0.6	12.0–14.0	<0.3	1.2–1.8	0.25–0.55	0.1–0.3

**Figure 1:**
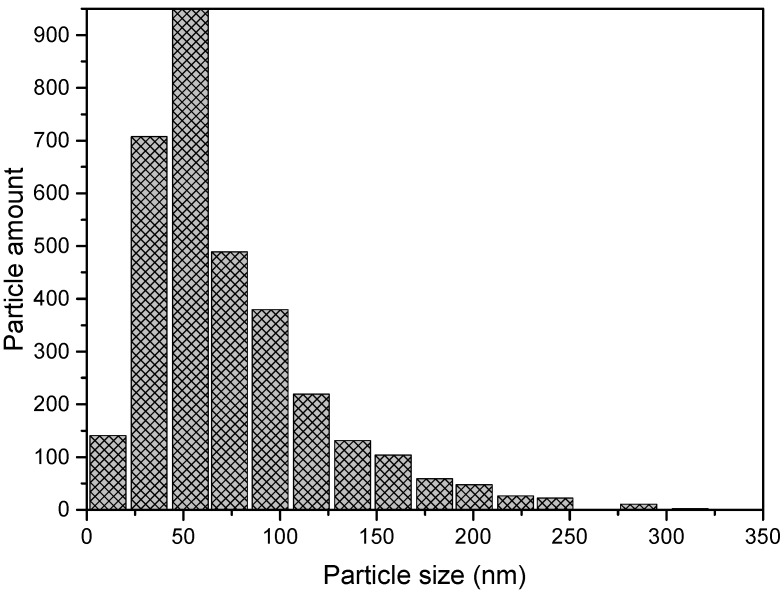
Particle size distribution of nanoscale Y_2_O_3_ powder.

This material in the form of flakes with the average size of 2–3 mm in length and 200–300 μm in thickness was obtained by casting the melt onto a rapidly rotated cooled massive disk. These flakes were pre-milled in a planetary ball mill (MTI LCC, Richmond, CA, USA) for 2 h in air to the state of a powder with particle sizes of up to 400 μm. This powder was mechanically mixed with different concentrations of commercial nanoscale Y_2_O_3_ powder (“Advanced Powder Technologies” LCC, Tomsk, Russia) with the average particle size of about 50 nm (Figure 1) and then was mechanically alloyed in a high-energy planetary ball mill (Fritsch GmbH, Idar-Oberstein, Germany) for 30 h in argon atmosphere.

During mechanical alloying the milling parameters such as rotation speed, time, and operation/standby intervals were varied in some range to obtain the most homogeneous powder. It was determined that the rotation speed and the working period of the mill may cause significant heating and agglomeration of the powder. Thus, these parameters were optimized to prevent such negative implications. On the other hand, the influence of the milling time on the particle size was investigated. The average particle size of the powder starts to reduce significantly at the beginning of the process and further mechanical alloying does not allow any significant reduction of the average particle size.

More details on the influence of the milling time on the particle size distribution in this powder system are provided in Ref. [19]. As a result, the milling time of 30 h at the rotation speed of 200 rpm and a 2 h/2 h operation/standby cycle were chosen as optimal parameters for mechanical alloying. The chemical composition of the obtained powders indicates that there are no significant changes of the yttria concentration or an appearance of any impurities during mechanical alloying (Table 2).

**Table 2:** Chemical composition of the powder after mechanical alloying with 0.3 wt% of yttria.

Element	Fe	Si	Mn	Cr	Ni	Mo	Nb	V	Y
Concentration, wt%	83.11	0.058	0.43	12.9	0.11	1.4	0.20	0.29	0.31

To obtain the data on the effect of the yttria concentration on the densification and other properties of the powders, two different concentrations of Y_2_O_3_ were added to the powder batches at the stage of mechanical alloying of 0.3 and 0.7 wt%, and the powder without yttria content was also used in the comparative analysis.

**Figure 2:**
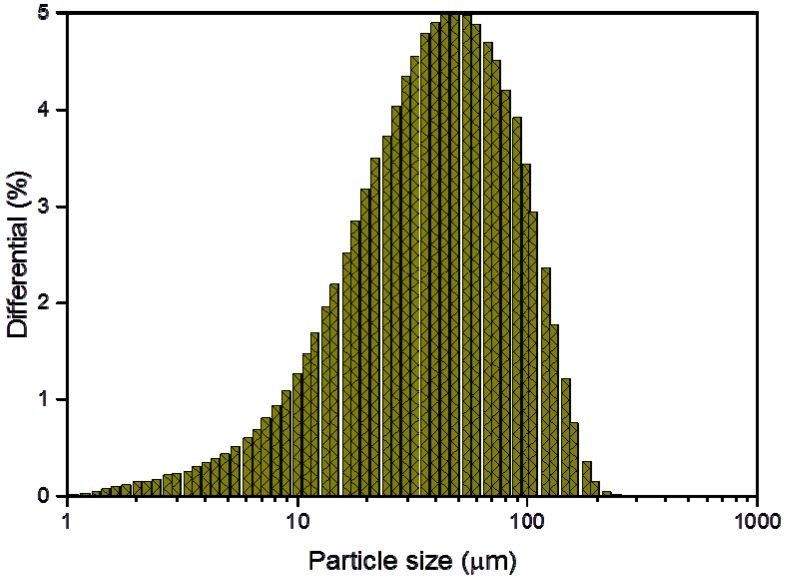
Typical particle size distribution of the obtained ODS steel powders.

The particle size analysis (Fritsch GmbH, Idar-Oberstein, Germany) shows that all powder batches with different yttria content have similar size distributions (Figure 2). The average particle size for all the batches was about 50 μm.

**Figure 3:**
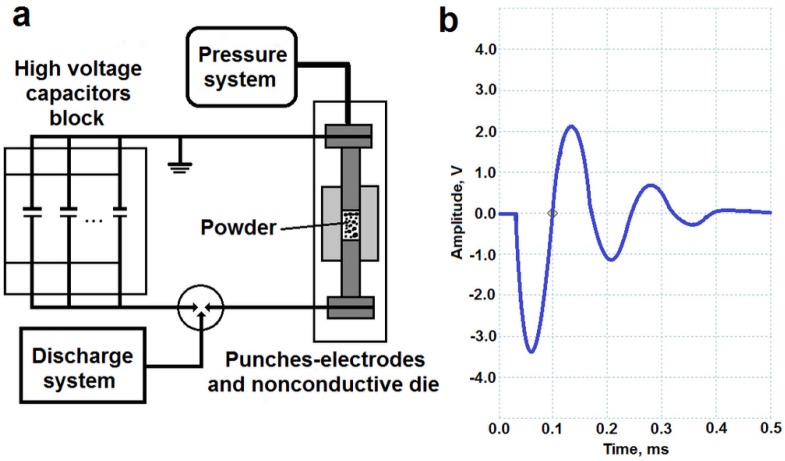
High voltage compaction principle scheme (**a**) and shape of the electric pulse (**b**).

The high voltage electric discharge compaction was provided by the Impulse-BM device (“Potok” LCC, Rostov-on-Don, Russia) (Figure 3a) which enables an electric discharge through the specimen with a voltage of up to 6 kV and a pressure of up to 10 atm. This equipment allows the application of the voltage of several kilovolts and the pressure of up to several atmospheres to a powder specimen. Depending on these parameters, a density of the electric current in the powder can be achieved up to 500 kA/cm^2^. The shape of the passing electric pulse is shown in Figure 3b and the time of the impact on the powder is up to several hundred milliseconds.

The consolidation was conducted for all the specimens using similar parameters. The powder was loaded in a non-conductive ceramic die with a rugged metal collar and Mo conductive electrodes were used as punches. The consolidation was held in air atmosphere and the initial mass of the powder filling was 5 g. The initial green density was about 55%. Three different pressure values, 170, 200, and 270 MPa, and five voltage values, 1.5, 2.0, 4.0, 4.2, and 4.4 kV, were used in the experiments. The obtained cylindrical samples were 10 mm in diameter and 10 to 15 mm in height.

Consolidated samples were mechanically polished and etched by 5 wt% nitric acid-alcohol mixture. The microstructure of the samples was investigated by means of optical microscopy. Microhardness data were obtained using a microhardness tester with 100 g loading and 15 s exposure. To obtain the data on the spatial density distribution inside the specimens’ volume, the processed samples were cut in layers in the axial direction. The final density was measured using three techniques—geometrically, with hydrostatic weighing, and with helium pycnometry. Final density for all the samples was within the range from 90% to 97.5%.

## 3. Results and Discussion

Consolidation conditions and properties of the samples are shown in Table 3. It should be noted that the consolidation regime for two of the samples (B4 and B7) was unstable, leading to the knockout of the powder though the gap between the punches and the die. The weight of the samples does not match the weight of the powder filling due to the surface roughness of the punches and the die whereby a part of the powder can spill out of the die.

It should be noted that HVEDC is a fast and a metastable process associated with high heating rates, with cooling rates, and with changes of electrical conductivity of the processed medium, and with other factors. In this regard, unstable regimes of consolidation may occur as indicated by a rapid increase of the conductivity of the consolidated medium, an avalanche temperature increase, liquid phase formation, knockout of the powder from the die, and by other effects. These facts should be taken into account when interpreting the experimental results since they significantly affect the structural and phase state of the processed material and its physical properties.

**Table 3:** Conditions of the compaction and properties of the samples.

Sample	Y_2_O_3_ Content, wt%	m_f_ *, g	m_s_ *, g	P, MPa	U, kV	Mode	*ρ*_rel._, %
B5	0.0	5.00	4.03	270	1.5	Stable	92.18
В7	0.0	5.00	3.48	200	4.4	Unstable	95.83
В8	0.0	5.00	4.32	200	4.0	Stable	96.09
B3	0.3	5.00	4.27	200	4.2	Stable	97.48
B4	0.3	5.00	4.50	270	4.0	Unstable	96.32
B6	0.3	5.00	4.72	270	2.0	Stable	97.04
B9	0.7	5.00	4.66	170	4.0	Stable	90.30
B10	0.7	5.00	4.83	170	4.2	Stable	93.33
B11	0.7	5.00	4.67	170	4.4	Stable	93.10

Notes: * m_f_—Mass of the powder filling; m_s_—Mass of the sintered sample.

**Figure 4:**
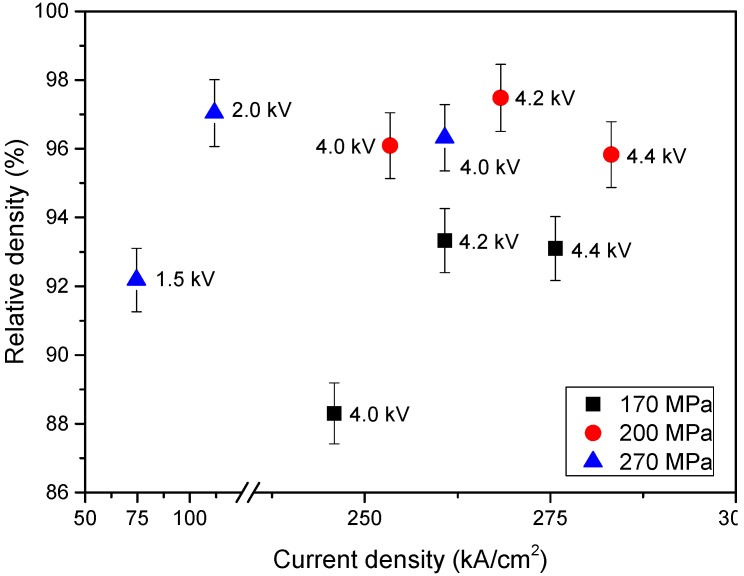
Samples’ relative density dependence on the density of the electric current.

First, the samples’ relative density dependence on the density of the electric current was determined (Figure 4). This approach clarifies how the transmitted electric power affects the compaction of the powder that allows the selection of optimal consolidation conditions. Since the electric current density depends on the resistivity of the powder at each moment of time, and it in turn depends on the pressure, the data was grouped according to the applied pressure. It is shown that the increasing pressure, in general, leads to the increasing final density. The samples, which were consolidated at 170 MPa pressure, have lower values of final density in comparison with the ones obtained at 200 or 270 MPa pressure. Also, the final density dependence on voltage was determined. An increase of the voltage values from 4.0 to 4.2 kV leads to the increasing relative density, but an insignificant decrease of the density was determined for the samples consolidated at 4.4 kV in comparison with the ones consolidated at 4.2 kV. This is valid for the whole range of used pressures. This effect may be associated with the occurrence of the local inhomogeneity of the electric current in the interparticle contacts due to which certain areas of the powder volume may include quite significant porosity. At the same time, samples that were consolidated at low values of the electric current density demonstrate quite high values of final density. This means that it is possible to obtain good-quality dense samples using quite low pressure and high voltage values and, at the same time, using high pressure but low voltage. Therefore, it should be noted that in the case of operating at low pressures, the conductivity of the powder composition may be insufficient for normal passing of the electric current. This may lead to an unstable consolidation process due to the high resistivity of the interparticle contacts resulting in the overheating of these areas, in the emergence of the electric breakdown between particles, and in the knockout of the powder. On the other hand, operating at high pressure values leads to better interparticle contacts and decreasing resistivity values of these contacts. This, in turn, leads to the lower energy release and heating of the powder which affects the sintering consolidation mechanism. It is clear that, using high pressures, it is possible to compact samples to high density values only by cold plastic deformation mechanisms, but strength and hardness of the produced samples may be insufficient. Also, quite strong die and punches are needed to realize such a process. Therefore, it is desirable to combine both mentioned mechanisms to obtain an acceptable product. As a result, 200 MPa and 4.2 kV consolidation conditions were chosen as optimal for the compaction of the ODS steel powder.

**Figure 5:**
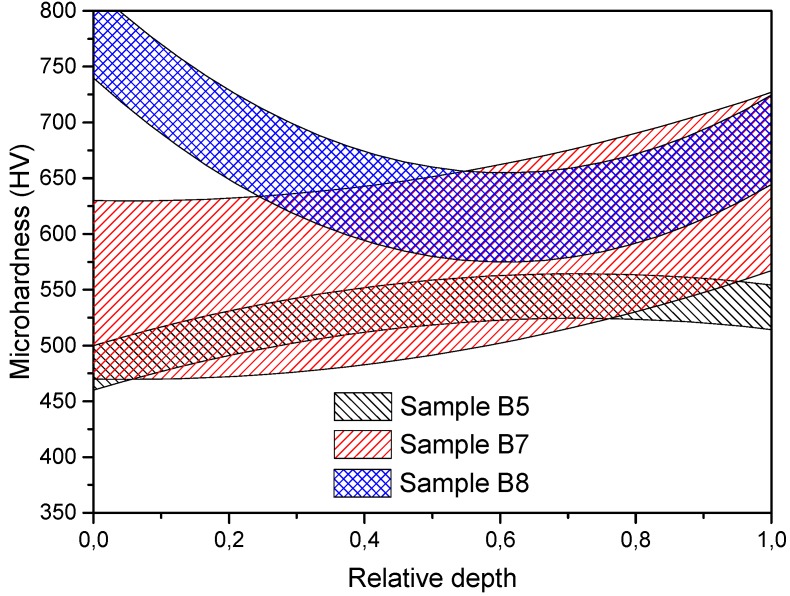
Axial microhardness distribution along samples’ depth without yttria addition.

**Figure 6:**
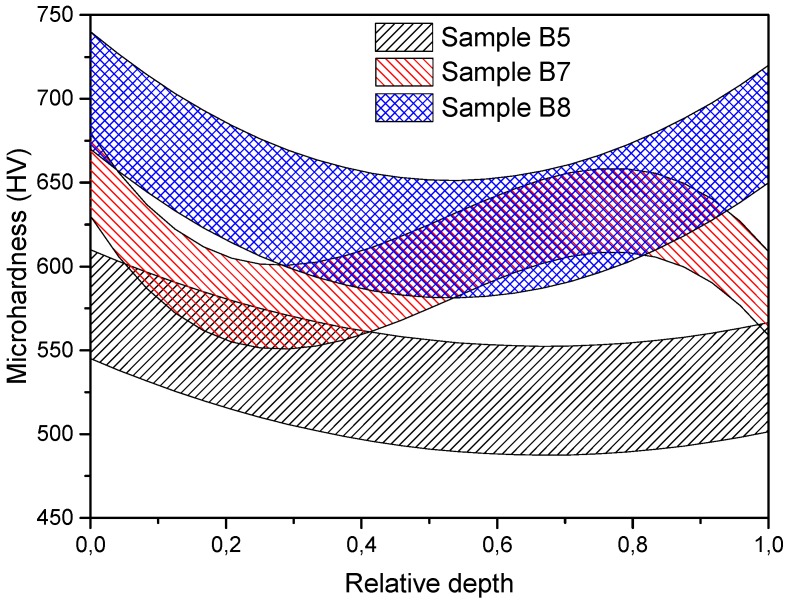
Radial microhardness distribution along samples’ depth without yttria addition.

The next step of the investigation was to determine the density and microhardness distribution in the volume of the samples. To obtain the above-mentioned data, the processed samples were measured using a microhardness tester (FM-800, Future-Tech, Kawasaki, Japan; 100 g loading for 10 s) in axial and radial directions with 50 µm steps between each measurement point. Measured values had quite significant scatter, so the final data were presented in the form of areas. Then the samples were cut in the axial direction into several layers to determine the spatial density distribution. Figure 5 shows the axial microhardness distribution of the samples without the yttria addition. Sample B5 (1.5 kV, 270 MPa) demonstrates quite uniform microhardness with insignificant decreasing at the edges and the average value of 525 HV. Sample B8 was consolidated using a higher voltage of 4.0 kV and a pressure of 200 MPa. The average microhardness of this sample is about 650 HV, but the edges of the sample have a higher microhardness in comparison to its center. This effect could be caused by non-uniform consolidation of the powder. During electric discharge the powder areas located closer to the punches can densify more intensively due to higher values of plastic deformation in comparison with the central part [20]. Another reason for this effect may be associated with high heating and cooling rates of these areas. After the electric discharge the punches rapidly cool the powder in these areas due to their high thermal conductivity, and a tempered structure may be formed. The microhardness values of approximately 775 and 675 HV at the edges of the sample correspond to the ones of the tempered structure. In the case of sample B5, the tempering had not occurred due to an insufficient discharge power. The consolidation conditions of sample B7 were unstable, which greatly affected the variation of the microhardness values and which is also observed in the radial microhardness distribution (Figure 6). It is clear that an unstable consolidation may cause a significant inhomogeneity of the structure. Other samples show similar microhardness distributions. Increasing hardness of the edges of the samples in the radial direction in this case may be present due to two factors. One of them is the emergence of the skin-effect in the surface layers of the samples due to the high-frequency discharge with the frequency of 6–7 kHz. This effect causes an additional heating of these areas and intensive plastic deformation. Another reason is the forming of the tempered structure due to high cooling rates in comparison with those of the central areas. In general, the average microhardness values correlate with the density of the samples: B5 has 92% relative density and B8 has 96%.

**Figure 7:**
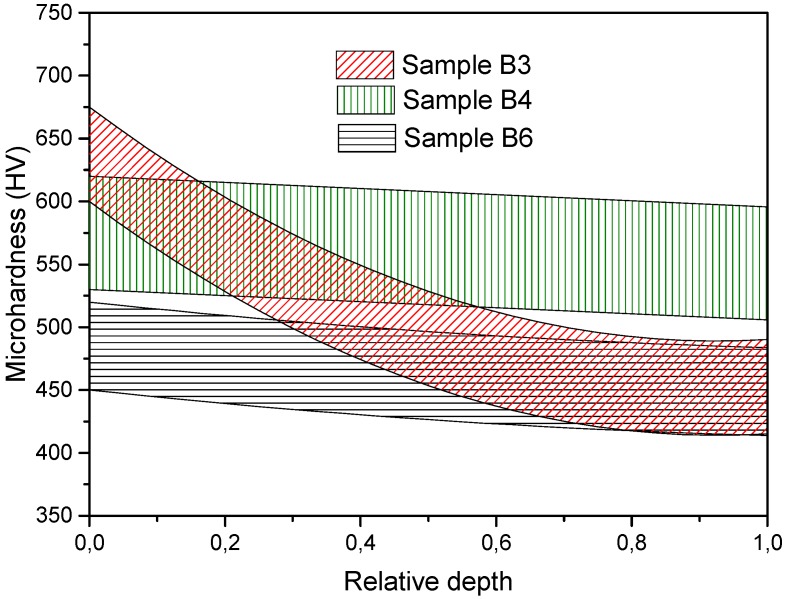
Axial microhardness distribution along samples’ depth with 0.3 wt% of yttria addition.

**Figure 8:**
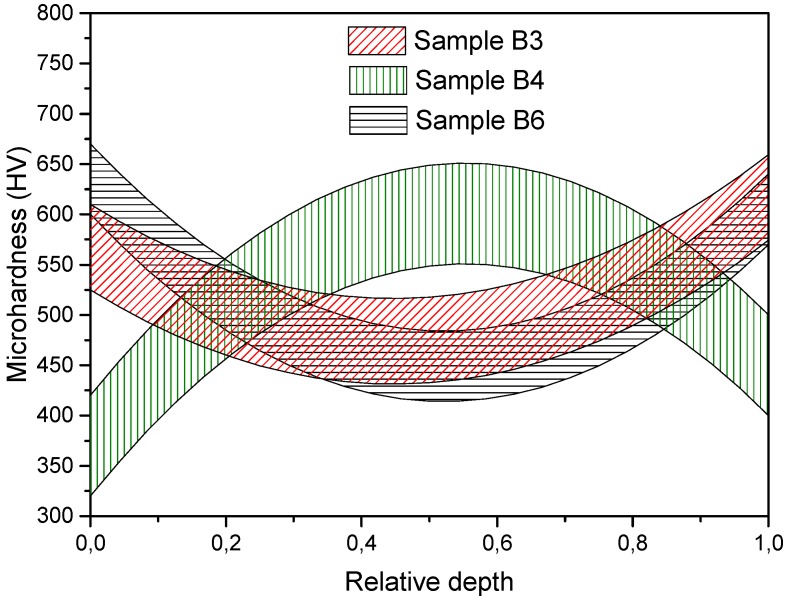
Radial microhardness distribution along samples’ depth with 0.3 wt% of yttria addition.

A similar pattern as described above was observed for the next group of the samples (Figure 7 and Figure 8). Samples B4 and B6 have uniform microhardness in the axial direction with the average values of 575 and 475 HV, respectively, but sample B3 has a significant difference in microhardness between the upper and lower edges which may be caused by a non-uniform pressing of the powder before electric discharge. Such effects may occur due to the friction of the powder against the walls of the die. The difference in the density distribution for sample B4 is also associated with an unstable consolidation regime. Other samples have the average microhardness values of 475 and 500 HV and density values of 97%. The strengthening effect of nanoparticles is a known phenomenon [21]. However, any significant dependence of the microhardness on the content of yttria had not been identified because of the rather small content of the oxide.

**Figure 9:**
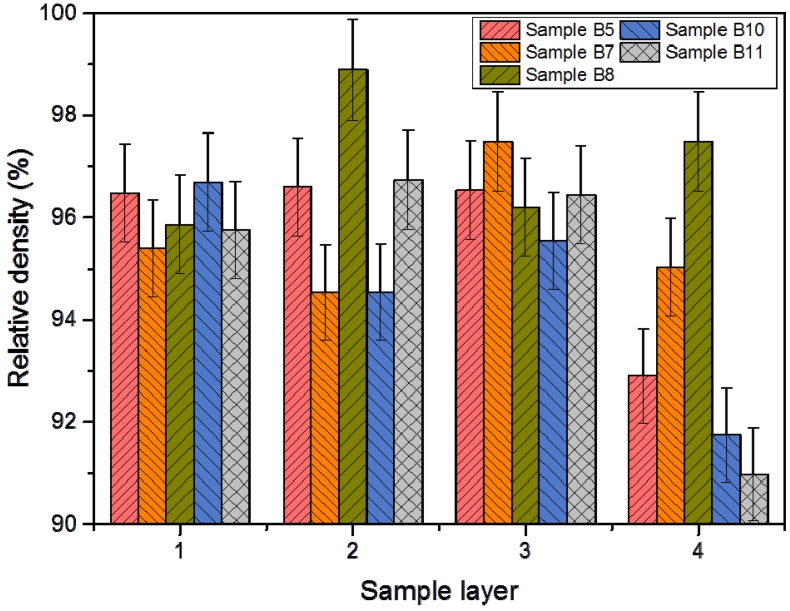
Axial density distribution along depth of the samples.

The axial density distribution of the consolidated samples is shown in Figure 9. The experimental data for the two groups of samples containing 0 wt% (samples B5, B7, B8) and 0.7 wt% of yttria (samples B10, B11) were obtained. It is clear that there is no stable density uniformity between layers of the processed samples. Therefore, on average, the difference from layer to layer is from 2% to 3%, which is not a significant enough value to determine any regular patterns of its distribution. There is a slight decrease in density for the layer adjacent to the lower punch for several samples due to the non-uniform pressing conditions before the electric discharge. Despite the fact that the pressing process is double-sided with a loosely attached die, there is some friction of the powder against the wall of the die during pressing. This leads to the non-uniform densification of the powder. Also, no correlation between density and microhardness distribution was observed. This may be due to the fact that different factors independently affect the microhardness and the density of the samples. The factors affecting microhardness may depend not only on the density but also on the phase composition, on the presence of impurities or defects, and on other factors.

**Figure 10:**
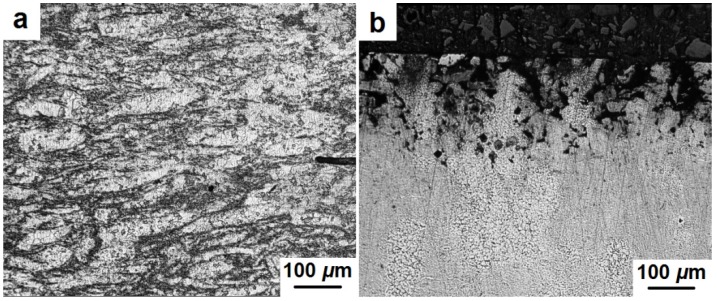
Microstructure of the center (**a**) and edge (**b**) of the consolidated samples.

Microstructure analyses of the consolidated samples were held to determine structure dependence on various factors of the compaction process such as applied pressure and cooling rate after electric discharge. The central area and the periphery of the samples were studied. Figure 10a shows a typical microstructure of the consolidated compacts in the center of the sample. It can be seen that the structure consists of some light color formations which may include agglomerated powder particles with a dark color fine structure between them. The size of the observed agglomerates is 20–50 μm along the minor axis and 100–200 μm along the major axis. The agglomerates are predominantly orientated in the direction perpendicular to the compaction axis of the sample, *i.e.*, they are flattened under pressure during the consolidation process. Figure 10b shows the microstructure at the edge of the sample. It is clear that the porous area reaches the depth of 100–150 μm and then a fairly dense structure is observed.

**Figure 11:**
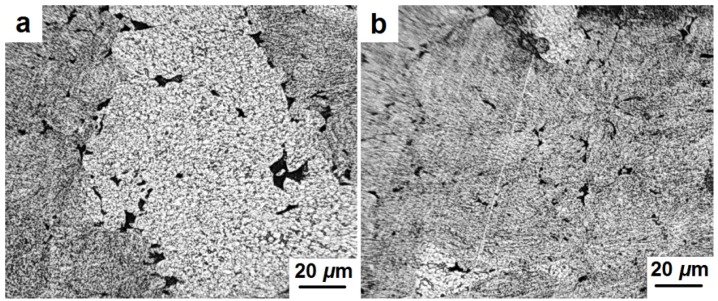
Microstructure of the center area (**a**,**b**) of the consolidated samples.

**Figure 12:**
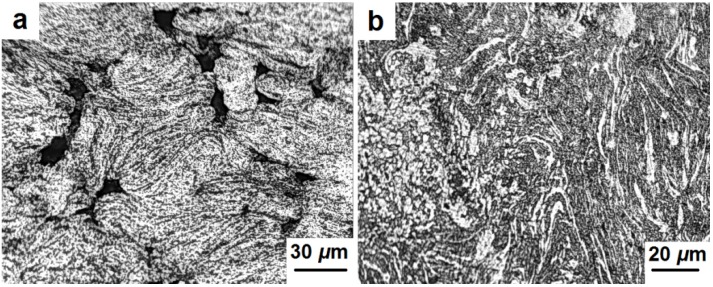
Microstructure of the edge (**a**) and side surface (**b**) of the consolidated samples.

Figure 11 and Figure 12 indicate microstructure differences between various areas of the samples’ volume (center, edge, and side surface). A more detailed examination of the samples’ surface allows distinguishing the grain structure and the presence of pores (Figure 11a,b). These pores are substantially elongated and located along grain boundaries. A non-typical structure was also observed primarily at the edge of the samples. Figure 12a shows a porous structure at the edge of the sample adjacent to the punches. Due to the rapid cooling the martensitic structure is formed in these areas. A different structure was observed at the edge of the sample adjacent to the side surface (Figure 12b). It is a mixture of ferrite and perlite with a distance between the perlite plates of around 1–2 μm. This structure could be formed as a result of intensive cooling of the side surface of the sample, but not as rapidly as in the case shown in Figure 12a.

## 4. Conclusions

Oxide dispersion-strengthened 13Cr-2Mo steel powder was successfully consolidated to near-theoretical density using high voltage electric discharge compaction. Cylindrical samples with relative density from 90% to 97% and dimensions of 10 mm in diameter and 10–15 mm in height were obtained.

It was found that high pressures lead to better interparticle contacts and to the decreasing resistivity of these contacts. This, in turn, leads to the lower energy release and heating of the powder which affects the sintering consolidation mechanism. It is clear that, using high pressures, it is possible to compact samples to high density values only by cold plastic deformation mechanisms; however, the strength and hardness of such samples may be insufficient. Therefore, it is necessary to combine both mentioned mechanisms to obtain an acceptable product. As a result, 200 MPa and 4.2 kV consolidation conditions were chosen as optimal for the compaction of ODS steel powder.

A slight increase of the microhardness at the edges of the samples in the axial and radial directions was observed. During electric discharge, the powder areas located in the vicinity of the punches can densify more intensively due to a higher degree of plastic deformation in comparison with the central parts of the processed specimens. Another reason for this effect may be associated with the high heating and cooling rates of these areas.

No correlation between density and microhardness distribution in the volume of the processed samples was observed. This may be due to the fact that different factors independently affect the microhardness and density of the samples.

It was shown that the edges of the samples adjacent to the punches have a martensitic structure due to rapid cooling in these areas. A different structure was observed at the edges adjacent to the side surface of the processed samples. It is a mixture of ferrite and perlite with a distance between the perlite plates of around 1–2 μm. This structure could be formed as a result of the intensive cooling of the side surface of the samples, but not as rapidly as in the locations of the martensitic phase.

In general, high voltage compaction is an acceptable method for the consolidation of ODS steels. This ultra-rapid process allows obtaining high density compacts, retaining the initial structure with minimal grain growth, and avoiding thermally activated phase transformations. Nevertheless, some heterogeneity of the structure may occur in the boundary layers of the processed samples due to thermal and electromagnetic effects. Therefore, the choice of the optimal parameters of consolidation is required for obtaining samples of acceptable quality.
